# Broad-spectrum resistance to *Bacillus thuringiensis* toxins by western corn rootworm (*Diabrotica virgifera virgifera*)

**DOI:** 10.1038/srep27860

**Published:** 2016-06-14

**Authors:** Siva R. K. Jakka, Ram B. Shrestha, Aaron J. Gassmann

**Affiliations:** 1 Department of Entomology, Iowa State University, Ames, IA 50011, USA.

## Abstract

The evolution of resistance and cross-resistance threaten the sustainability of genetically engineered crops that produce insecticidal toxins derived from the bacterium *Bacillus thuringiensis* (Bt). Western corn rootworm, *Diabrotica virgifera virgifera* LeConte, is a serious pest of maize and has been managed with Bt maize since 2003. We conducted laboratory bioassays with maize hybrids producing Bt toxins Cry3Bb1, mCry3A, eCry3.1Ab, and Cry34/35Ab1, which represent all commercialized Bt toxins for management of western corn rootworm. We tested populations from fields where severe injury to Cry3Bb1 maize was observed, and populations that had never been exposed to Bt maize. Consistent with past studies, bioassays indicated that field populations were resistant to Cry3Bb1 maize and mCry3A maize, and that cross-resistance was present between these two types of Bt maize. Additionally, bioassays revealed resistance to eCry3.1Ab maize and cross-resistance among Cry3Bb1, mCry3A and eCry3.1Ab. However, no resistance or cross-resistance was detected for Cry34/35Ab1 maize. This broad-spectrum resistance illustrates the potential for insect pests to develop resistance rapidly to multiple Bt toxins when structural similarities are present among toxins, and raises concerns about the long-term durability of Bt crops for management of some insect pests.

Western corn rootworm, *Diabrotica virgifera virgifera* LeConte (Coleoptera: Chrysomelidae), is one of the most economically important insect pests of maize in the United States^1^. Crop losses from this pest are primarily attributed to the larval feeding on roots, which reduces yield and can complicate harvest if maize plants lodge (i.e., fall over)^2^,^3^,^4^. Managing western corn rootworm has been a challenge, in part, because this pest has developed resistance to several management approaches^5^,^6^,^7^. Since the 1980s, the cost to farmers from western corn rootworm, both in terms of crop losses and management inputs, is estimated to meet or exceed one billion US dollars per year^1^.

Genetically engineered maize hybrids producing insecticidal toxins derived from the bacterium *Bacillus thuringiensis* (Bt) were made available for the management of western corn rootworm beginning in 2003. The first generation of Bt hybrids introduced to protect maize plants from larval rootworm injury produced Bt toxins singly, and these included Cry3Bb1, mCry3A, and Cry34/35Ab1^8^,^9^. The cultivation of Bt maize, along with Bt cotton, has provided several benefits to farmers and the environment, including reduced harm to non-target organisms compared with conventional insecticides, reductions in the use of conventional insecticides and increased profits for farmers^10^,^11^,^12^,^13^. However, the evolution of pest resistance threatens to diminish these benefits.

Recently, field-evolved resistance by western corn rootworm to Cry3Bb1 maize and mCry3A maize was documented in multiple Midwestern states, and cross-resistance has been identified between these Bt toxins^14^,^15^,^16^. To mitigate the effects of Bt-resistant populations of western corn rootworm and to delay additional cases of resistance, pyramided maize hybrids, with multiple Bt toxins targeting corn rootworm, were made available to farmers. Currently, there are four Bt toxins that are used to manage western corn rootworm: Cry3Bb1, Cry34/35Ab1, mCry3A, and eCry3.1Ab, and these were registered by the US EPA in 2003, 2005, 2006 and 2012, respectively^8^. Additionally, these toxins have been used to produce three types of pyramided maize targeting western corn rootworm: Cry3Bb1 + Cry34/35Ab1, mCry3A + Cry34/35Ab1, mCry3A + eCry3.1Ab^8^. However, the efficacy of these pyramided Bt maize hybrids may be influenced by resistance to the individual Bt toxins within pyramids and by cross-resistance between Bt toxins within a pyramid^17^, with cross-resistance occurring when resistance to one Bt toxin reduces susceptibility to another Bt toxin^18^. Cross-resistance among Bt toxins can be due to similarities in the mode of action and several studies have found cross-resistance between Bt toxins^19^,^20^,^21^,^22^,^23^.

In cases where western corn rootworm have developed resistance to mCry3A and Cry3Bb1^15^,^16^, pyramided Bt maize hybrids primarily rely on either Cry34/35Ab1 or eCry3.1Ab to reduce larval feeding injury^24^. However, the presence of cross-resistance between Bt toxins within a pyramid would diminish the efficacy of pyramided maize against western corn rootworm. Past studies indicate an absence of cross-resistance between Cry3Bb1 and Cry34/35Ab1^14^,^15^,^16^,^25^. However, structural similarities between mCry3A and eCry3.1Ab suggest the presence of cross-resistance between these Bt toxins, and recent research has found evidence of cross-resistance^17^,^26^,^27^.

Moreover, to the extent that pyramided Bt hybrids are planted to manage Cry3Bb1-resistant and mCry3A-resistant populations of western corn rootworm, it is likely that western corn rootworm populations will experience intense selection pressure to develop resistance to eCry3.1Ab and Cry34/35Ab1 toxins. Laboratory selection experiments indicate that western corn rootworm has the ability to develop resistance to all currently commercialized Bt toxins following three to seven generations of selection^28^,^29^,^30^,^31^. Similarly, field-evolved resistance to Cry3Bb1 maize was observed in western corn rootworm populations collected from fields that had been planted to Cry3Bb1 maize continuously for 3 to 7 years^14^. The vulnerability of Bt maize to resistance by western corn rootworm necessitates an understanding of the patterns of resistance and cross-resistance so that more effective approaches can be developed to manage resistance for this economically important pest insect. In the present study, we tested patterns of resistance and cross-resistance for western corn rootworm against all commercially available Bt toxins (Cry34/35Ab1, Cry3Bb1, mCry3A and eCry3.1Ab). Our results reveal resistance to Cry3Bb1 maize, mCry3A maize, and eCry3.1Ab maize for populations of western corn rootworm from fields with high levels of feeding injury to Cry3Bb1 maize, and cross-resistance among these Cry3 Bt toxins.

## Results

**Figure 1:**
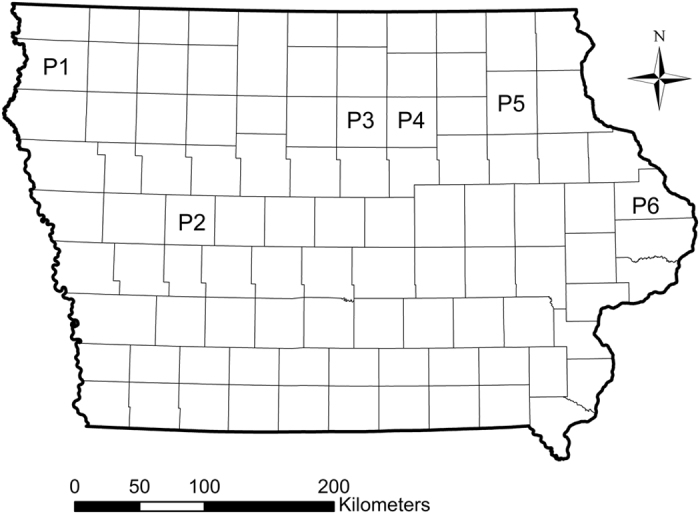
Distribution of fields sampled in Iowa, USA during 2012 that were then used in subsequent bioassays. Alphanumeric symbols represent the location of each field and are accurate to the level of the individual county, with counties represented by the sub-divisions within Iowa. Based on the 0 to 3 node injury scale of Oleson *et al.*^56^, the level of root injury to Cry3Bb1 maize observed in each field was (mean ± SE): P1 = 2.1 ± 0.20, P2 = 2.3 ± 0.12, P3 = 2.7 ± 0.10, P4 = 3.0 ± 0.00, P5 = 1.5 + 0.28, P6 = 2.6 ± 0.13. The map was generated using ArcGIS 10.0 (Esri, Redlands, California).

Plant-based bioassays were conducted to test for resistance and cross-resistance to maize hybrids producing rootworm-active Bt toxins. In total, five Bt-susceptible control populations were tested alongside six field populations that were sampled from fields where greater than one node of root injury to Cry3Bb1 maize was observed. Field populations were distributed throughout northern and central Iowa (Fig. 1). Root injury ratings to Cry3Bb1 maize for each field used in this study were (mean number of nodes ± SE): P1 = 2.1 ± 0.20, P2 = 2.3 ± 0.12, P3 = 2.7 ± 0.10, P4 = 3.0 ± 0.00, P5 = 1.5 ± 0.28, P6 = 2.6 ± 0.13. For each combination of population by hybrid tested with plant-based bioassays, the mean for proportion survival, associated standard error of the mean and sample size were calculated (Table S1).

**Figure 2:**
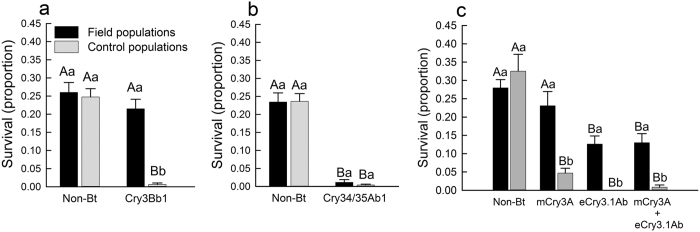
Survival of western corn rootworm larvae from field populations and control populations on (**a**) Cry3Bb1 maize and its non-Bt near isoline, (**b**) Cry34/35Ab1 maize and its non-Bt near isoline, and (**c**) mCry3A maize, eCry3.1Ab maize, maize pyramided with mCry3A + eCry3.1Ab, and the non-Bt near isoline to these Bt maize hybrids. Bar heights represents sample means among field populations (n = 6) and control populations (n = 5). Error bars are the standard error of the mean. Capital letters indicate pairwise differences between means for a population type (e.g. control populations) within an individual graph, and lower case letters indicate pairwise differences between population types (i.e., field populations versus control populations) within a hybrid (e.g., Cry3Bb1 maize).

Analysis of variance for larval survival revealed a significant interaction between population type and maize hybrid (F = 17.46; df = 7,63; P < 0.0001). For all three non-Bt maize hybrids, larval survival did not differ between field populations and control populations, whereas significantly greater larval survival was observed for field populations, relative to control populations, on Cry3Bb1 maize, mCry3A maize, eCry3.1Ab maize, and maize pyramided with mCry3A and eCry3.1Ab hybrids, indicating resistance to these Bt hybrids (Fig. 2). Survival of larvae from field populations on Cry3Bb1 maize and mCry3A maize did not differ significantly from the non-Bt near isoline (Fig. 2a,c). However, larval survival of field populations on eCry3.1Ab maize and maize pyramided with eCry3.1Ab and mCry3A was significantly lower than survival on the non-Bt near isoline (Fig. 2c). By contrast, larval survival of field populations and control populations was not significantly different on Cry34/35Ab1 maize hybrids, and was significantly lower compared to the non-Bt isoline (Fig. 2b), pointing to a lack of resistance to Cry34/35Ab1 maize.

**Figure 3:**
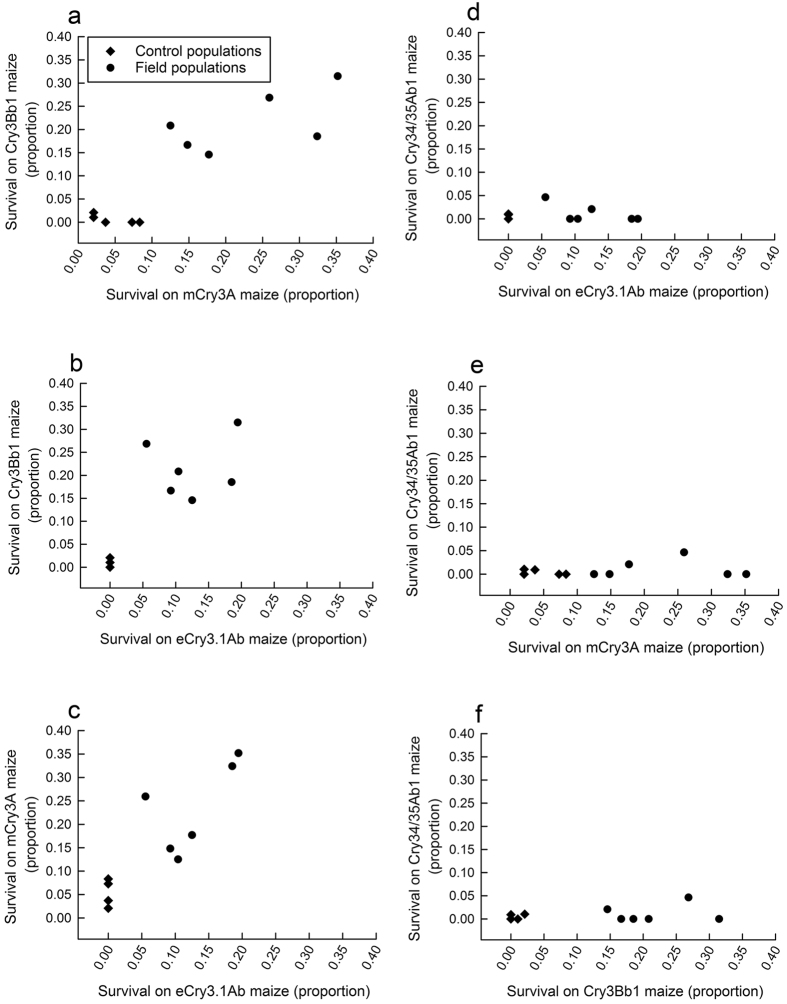
Correlations among populations for survival on Bt maize hybrids. Diamonds represent control populations and circles represent field populations. The types of Bt maize compared, and associated correlation coefficients are (**a**) Cry3Bb1 maize vs. mCry3A maize (r = 0.88; df = 9; P = 0.0004), (**b**) Cry3Bb1 maize vs. eCry3.1Ab maize (r = 0.83; df = 9; P = 0.002), (**c**) mCry3A maize vs. eCry3.1Ab maize (r = 0.89; df = 9; P = 0.0003), (**d**) Cry34/35Ab1 maize vs. eCry3.1Ab maize (r = −0.07; df = 9; P = 0.83), (**e**) Cry34/35Ab1 maize vs. mCry3A maize (r = 0.20; df = 9; P = 0.55), and (**f**) Cry34/35Ab1 maize vs. Cry3Bb1 maize (r = 0.31; df = 9; P = 0.36).

Results from correlation analyses revealed a significant positive relationship among populations for larval survival on Cry3Bb1, mCry3A, and eCry3.1Ab maize hybrids, indicating the presence of cross-resistance among these Bt toxins (Fig. 3). A positive correlation existed for larval survival on Cry3Bb1 maize versus mCry3A (r = 0.88; df = 9; P = 0.0004) (Fig. 3a) and eCry3.1Ab maize (r = 0.83; df = 9; P = 0.002) (Fig. 3b). A positive correlation for larval survival also was found between mCry3A maize and eCry3.1Ab maize (r = 0.89; df = 9; P = 0.0003) (Fig. 3c). By contrast, no significant correlations were detected between Cry34/35Ab1 maize and either Cry3Bb1 maize, mCry3A maize, or eCry3.1Ab maize (P > 0.35 in all cases) (Fig. 3d–f), pointing to a lack of cross-resistance between Cry34/35Ab1 and the other types of Bt maize tested. Additionally, there was no significant correlation among populations for survival on non-Bt maize hybrids (P > 0.10 in all cases), indicating that survival was similar among populations in the absence of Bt toxins. These correlation results indicate cross-resistance among populations for survival on Cry3Bb1 maize, mCry3A maize, and eCry3.1Ab maize, but a lack of cross-resistance between Cry34/35Ab1 maize and any of the other Bt toxins tested.

Data on larval development, as measured by the proportion of larvae that reached the third and final instar, indicated no significant difference for field populations on non-Bt maize compared to either mCry3A maize, eCry3.1Ab maize or maize pyramided with mCry3A and eCry3.1Ab (Table 1). However, for control populations on both mCry3A maize and maize with eCry3.1Ab, there were associated developmental delays, because a significantly lower proportion of third instar larvae were found on mCry3A maize and maize with eCry3.1Ab compared to non-Bt maize (Table 1). For field populations, significantly fewer third instar larvae were recovered from Cry3Bb1 maize compared to the non-Bt near isoline, indicating delayed development of field populations on Cry3Bb1 maize. By contrast, developmental rate for control populations did not differ between Cry3Bb1 maize and the non-Bt near isoline, although the small sample size for control populations on Cry3Bb1 maize likely diminished the statistical power of this comparison (Table 1). For both field populations and control populations the proportion of third instar larvae on Cry34/35Ab1 maize was significantly less than on the non-Bt near isoline (Table 1).

## Discussion

The development of insect resistance and cross-resistance to Bt toxins can reduce the effectiveness of Bt crops for managing insect pests and represents a serious threat to the development of more sustainable pest management practices^32^. The field-collected populations of western corn rootworm in our study displayed resistance to Cry3Bb1 maize, mCry3A maize and eCry3.1Ab maize, as demonstrated by elevated survival on these types of Bt maize compared to known Bt-susceptible control populations, but an absence of resistance to Cry34/35Ab1 maize (Fig. 2). No difference in either larval survival or developmental rate was present for field populations on mCry3A maize compared to non-Bt maize, suggesting complete resistance to this Bt toxin (Fig. 2c, Table 1). By contrast, for these field populations, resistance to both Cry3Bb1 maize and eCry3.1Ab maize appeared to be incomplete. Although survival did not differ for field populations on Cry3Bb1 maize versus the non-Bt near isoline, field populations displayed delayed development on Cry3Bb1 maize compared to non-Bt maize (Fig. 2a, Table 1). For field population on eCry3.1Ab maize, larval development did not differ between eCry3.1Ab maize and the non-Bt near isoline, but survival was significantly lower on eCry3.1Ab maize compared to non-Bt maize (Fig. 2c, Table 1). Since the initial identification of western corn rootworm resistance to Cry3Bb1 maize in 2011, the magnitude of resistance observed within field populations has increased and cross-resistance to mCry3A maize has been documented^14^,^15^,^25^. Factors affecting the evolution of Bt resistance in western corn rootworm may include the amount of Bt maize in the landscape, a lack of a high dose produced by Bt maize targeting corn rootworm, failure to follow refuge requirements, a lack of fitness costs accompanying Bt resistance, and insufficient use of alternative pest management practices^9^,^33^.

Our study detected cross-resistance among Bt toxins Cry3Bb1, mCry3A, and eCry3.1Ab (Fig. 3). Past research has found evidence for cross-resistance between Cry3Bb1 and mCry3A toxins, and more recent work also found evidence of cross-resistance among eCry3.1Ab, Cry3Bb1 and mCry3A^15^,^16^,^26^. Bt toxin eCry3.1Ab was generated by exchanging the structural domains of two Bt Cry toxins. Many Bt Cry toxins, including Cry3 toxins, are classified as three domain toxins, and each domain contributes to the mode of action. Domain I is involved in pore formation, domain II is involved in binding specificity to receptors on the membrane of the midgut, and domain III is involved in Bt toxin stability and binding specificity^34^,^35^. Bt toxin eCry3.1Ab was engineered by replacing domain III of the coleopteran-active mCry3A toxin with domain III of the lepidopteran-active Cry1Ab toxin^36^, which increased efficacy against western corn rootworm larvae compared to mCry3A^37^. The eCry3.1Ab and mCry3A Bt toxins have the same domain II^36^,^38^, which is responsible for binding specificity^39^. Previous binding studies have detected two binding sites for mCry3A and one binding site for eCry3.1Ab on the gut membrane of western corn rootworm^27^. It may be the case that eCry3.1Ab shares one of the mCry3A binding sites, and this shared binding site may contribute to the observed pattern of cross-resistance.

Studies characterizing Bt resistance by pest insects often emphasize the role of modification in the binding sites of Bt toxins^40^,^41^,^42^, and the alteration of gut membrane receptors is often reported as a mechanism of Bt resistance^43^,^44^. The development of cross-resistance among Bt toxins in target insects can depend on shared binding sites in the insect’s midgut and amino acid similarities between Bt toxins^20^,^45^. Complete information on the interaction of Cry3Bb1, mCry3A, eCry3.1Ab, and Cry34/35Ab1 with gut membrane receptors of western corn rootworm has yet to be reported. However, amino acid sequences are more similar among Cry3Bb1, mCry3A, and eCry3.1Ab than between any of these toxins and Cry34/35Ab1^17^. These patterns of amino acid similarities may help explain western corn rootworm cross-resistance among Cry3Bb1, mCry3A, and eCry3.1Ab. Similar to our study, Huang *et al.*^46^ reported the development of low levels of cross-resistance in Cry1F-resistant fall armyworm, *Spodoptera frugiperda* (J. E. Smith) to Cry1A.105, but not to Cry2Ab2 or Vip3A toxins. Bt toxin Cry1A.105 is a chimeric toxin composed of domain I and domain II of Cry1Ac, and a domain III of Cry1F^47^. Shared binding sites in fall armyworm also were reported between Cry1A.105 and Cry1F but not with Cry2Ab2^48^, which is consistent with the hypothesis that alterations in shared binding sites represent a mechanism of cross-resistance among Bt toxins.

Current approaches to resistance management for Bt crops, enacted by the US Environmental Protection Agency, promote the use of refuges and planting of pyramided Bt crops to delay the evolution of resistance^9^. Ideally, pyramids contain two or more efficacious Bt toxins that kill the same pest insect but have different modes of action, making it difficult for a pest population to develop resistance to both toxins^17^,^49^. In such cases, it is hypothesized that a pest population will only develop resistance to a pyramided Bt crop through the simultaneous evolution of resistance at two independent loci, with each locus conferring resistance to one Bt toxin, and that the likelihood of this occurring is low^41^,^49^,^50^,^51^,^52^,^53^. However, the efficacy of pyramided Bt crops to delay resistance may be diminished by either prior exposure of insect populations to one or both of the Bt toxins in a pyramid or by the existence of cross-resistance between Bt toxins^19^,^49^. Our study found western corn rootworm resistance to Cry3Bb1 and mCry3A, which may reduce the efficacy of pyramided Bt maize hybrids producing Cry3Bb1+ Cry34/35Ab1 or mCry3A + Cry34/35Ab1. In addition, we found cross-resistance between mCry3A and eCry3.1Ab, which may lead to increased root injury and resistance development for Bt maize pyramided with mCry3A + eCry3.1Ab. A similar reduction in efficacy of Bt cotton pyramided with Cry1Ac and Cry2Ab was observed in cotton bollworm *Helicoverpa zea* Boddie^54^. Given the current pattern of Bt resistance and cross-resistance in western corn rootworm, it appears likely that Cry3Bb1-resistant western corn rootworm populations in fields planted to pyramided maize (i.e., Cry3Bb1 + Cry34/35Ab1, mCry3A + Cry34/35Ab1, and mCry3A + eCry3.1Ab) will experience strong selection for resistance to Cry34/35Ab1 and eCry3.1Ab, which threatens to further compromise the efficacy of currently commercialized pyramided Bt maize hybrids targeting western corn rootworm.

Greater adoption of integrated pest management practices by farmers may reduce the rate of Bt resistance evolution in western corn rootworm by decreasing the intensity of selection for resistance^55^. Although planting of pyramided hybrids may mitigate the effects of western rootworm resistance to Cry3Bb1 maize by diminishing the level of injury, the resistance management benefit of these pyramids is likely compromised by the presence of resistance and cross-resistance among Cry3 Bt toxins^24^. To delay additional instances of Bt resistance by western corn rootworm greater diversification of management approaches likely will be essential.

## Methods

This study aimed to evaluate patterns of resistance and cross-resistance to rootworm-active Bt toxins in western corn rootworm populations collected from fields in Iowa, USA. Fields of Cry3Bb1 maize were visited during 2012 in response to reports of root injury received from farmers, crop consultants and regional agronomists. The presence of Cry3Bb1 maize within the field was confirmed with ELISA strips (Envirologix, Portland, Maine). Roots were sampled to quantify root injury and adult western corn rootworm were collected to obtain eggs for subsequent plant-based bioassays, following Gassmann *et al.*^15^. Briefly, within each field visited, roots (10 to 13) were collected every two meters from two parallel transects that were 15 m apart. Root injury was scored based on the 0.0 to 3.0 node injury scale^56^. A threshold of one node or more of root-injury was used to classify fields as having greater than expected feeding injury by western corn rootworm^57^. The location of each field was recorded using a global positioning system (GPS) (Legend HCX; Garmin International, Inc. Olathe, Kansas), and these locations were mapped by plotting GPS coordinates in Google Earth (Google, Inc., Mountain View, California). Locations were then transferred manually, accurate to the level of an individual county, to a map of Iowa generated in ArcGIS 10.0 (Esri, Redlands, California) using data layers provided by the Iowa Department of Natural Resources (Fig. 1).

From each field, ca. 300 adult western corn rootworm were collected and brought to the laboratory to collect eggs following Gassmann *et al.*^14^. Each population was held individually in a cage within a biological incubator (25 °C; 16/8 L/D) and eggs (N = 3,000 to 20,000) were collected from each population. Eggs were stored at 4 °C within walk-in chamber for a least 5 months to break diapause, with the resulting larvae used for plant-based bioassays to measure susceptibility to Bt maize.

Field populations were evaluated alongside Bt-susceptible control populations. Eggs from five control populations were provided by the United States Department of Agriculture’s North Central Agricultural Research Laboratory in Brookings, South Dakota. Control populations were brought into the laboratory culture before 2003, which marks the first year of commercial cultivation for Bt maize targeting corn rootworm. Thus, control populations never experienced selection for Bt resistance and remain susceptible to Bt toxins. The year that control populations were collected and the site of collection were: (1) 1995 Phelps Co., NE; (2) 1995 Potter Co., SD; (3) 1996 York Co., NE; (4) 2000 Centre Co., PA; (5) 2000 Finney Co., KS.

Five Bt maize hybrids and three non-Bt maize hybrids were used to conduct plant-based bioassays following Gassmann *et al.*^15^. Hybrids included Cry3Bb1 maize (event 88017) and its non-Bt near isoline (Monsanto Co., St. Louis, MO), and Cry34/35Ab1 maize (event DAS-59122-7) and its non-Bt near isoline (Dow AgroSciences, Indianapolis, IN). Additionally, mCry3A maize (event MIR604), eCry3.1Ab maize (event MIR5307), maize pyramid with mCry3A and eCry3.1Ab (event MIR5307 and event MIR604) and a non-Bt near isoline to these hybrids were tested (Syngenta, Basel, Switzerland). None of the seed used in bioassays contained a pesticidal seed treatment, but all seed was washed with 10% bleach to remove traces of pesticide that may have been present from seed storage or handling prior to arriving at Iowa State University, following Gassmann *et al.*^14^. Maize plants were grown in the greenhouse, individually, in 1 L containers, following Gassmann *et al.*^14^. Plants were grown until five to six fully formed leaves were present (i.e., V5 to V6 stage; ca. 4 weeks).

Single-plant bioassays with maize hybrids, following Gassmann *et al.*^15^, were conducted to evaluate resistance and cross-resistance to Bt maize hybrids by western corn rootworm. To obtain larvae for assays, eggs from field populations and control populations were removed from 4 °C, washed, and kept in an incubator (25 °C, 60% RH, 16/8 L/D). First instar larvae (less than 24 hours old) were placed on nodal roots of plants of each maize hybrid using a fine brush. A thin layer of soil was used to cover the exposed roots. For each of the 11 populations, either eight (N = 6) or nine (N = 5) maize plants from each of the eight hybrids received 12 larvae, with some exceptions that arose because of the limited availability of plants. Specifically, one control population had only four replications for all hybrids except Cry3Bb1 maize and the non-Bt near isoline, one field population had only four replications of Cry3Bb1 maize and its non-Bt near isoline, and one control population had only seven replicates for Cry3Bb1 maize. In total, for the 11 populations, 710 single-plant bioassays were run and used 8,520 larvae. After larvae were placed on roots, a barrier (tangle-foot, Contech, British Columbia, Canada) was applied to the lip of each 1 L container to prevent larval movement between bioassay containers.

Bioassay plants were held in an incubator (24 °C, 60% RH, 16/8 L/D) and watered as needed, with plants randomized weekly. Larvae fed on the roots of the bioassay plants for 17 days in the incubator before being transferred to Berlese funnels. Roots and soil were kept on Berlese funnels for 4 days to collect surviving larvae in glass vials containing 85% ethanol. The larvae recovered were counted and their instar was determined based on head capsule width following Hammack *et al.*^58^. For each bioassay plant, proportion survival was calculated as the number of larvae that survived divided by the number of larvae originally placed onto roots. Bioassays were conducted between July 2013 and February 2014. All bioassays conducted with an individual population were repeated if larval recovery was less than 12.5 percent on any of the three non-Bt hybrids. Three out of six populations collected from the field and two out of five control populations were repeated because larval recovery from the initial bioassays was too low.

Data on proportion survival per bioassay container were analyzed with a mixed-model analysis of variance (ANOVA) (PROC MIXED) in SAS^59^. Data were transformed by the arcsine of square root to improve normality of the residuals and homogeneity of variance. In the analysis, population type (field population vs. control population), maize hybrid (Cry3Bb1 maize, non-Bt near isoline to Cry3Bb1 maize, Cry34/35Ab1 maize, non-Bt near isoline to Cry34/35Ab1 maize, mCry3A maize, eCry3.1Ab maize, eCry3.1Ab + mCry3A maize, and non-Bt near isoline to mCry3A/eCry3.1Ab hybrids), and the interaction between population type and maize hybrid were used as fixed factors. Population nested within population type, and the interaction between maize hybrid and population nested within population type were used as random factors. Because a significant interaction was present between population type and hybrid, pairwise comparisons were made within each hybrid family to understand the nature of this interaction. Within each hybrid family, all possible pairwise comparisons were made among hybrids within a population type (e.g., control populations), and between population types within each hybrid (e.g., Cry3Bb1 maize). In total, 24 pairwise comparisons were conducted (four comparisons within Cry3Bb1 maize and its non-Bt near isoline (Fig. 2a), four comparisons within Cry34/35Ab1 maize and its non-Bt near isoline (Fig. 2b), and 16 comparisons within mCry3A maize, eCry3.1Ab maize, eCry3.1Ab + mCry3A maize, and non-Bt near isoline (Fig. 2c). Pairwise comparisons were made with a significance level of *P* < 0.0021 based on a Dunn-Šidák correction for 24 comparisons^60^.

An evaluation of cross-resistance among Cry toxins in Bt maize hybrids was conducted based on correlation analysis (PROC CORR in SAS). Data used in the correlation analysis were the proportion survival for each of the 11 populations on the four single-toxin Bt hybrids (i.e., Cry3Bb1 maize, mCry3A maize, eCry3.1Ab maize and Cry34/35Abl maize) and the three non-Bt near isolines. For each combination of population by hybrid, we calculated proportion survival by taking the average for proportion survival in each of the individual experimental replicates (i.e., each of the single-plant bioassays for a given combination of population by hybrid). In every correlation analysis, a Pearson correlation coefficient was calculated and tested for statistical significance against the null hypothesis of ρ = 0, with the alternative hypothesis ρ ≠ 0.

Larval developmental rate was evaluated by calculating the proportion of larvae that reached the third and final instar (i.e., number of third instar larvae recovered divided by the total number of larvae recovered). To test for a developmental delay on Bt corn, the mean proportion of third instar larvae on a Bt hybrid (e.g., Cry3Bb1 corn) was compared to the respective non-Bt near isoline with a t-test (PROC TTEST in SAS). This was done for both control populations and problem field populations. In each comparison, homogeneity of variance was evaluated with an F test, and when the variance was unequal a Satterthwaite correction was applied (PROC TTEST).

## Additional Information

**How to cite this article**: Jakka, S. R. K. *et al.* Broad-spectrum resistance to *Bacillus thuringiensis* toxins by western corn rootworm (*Diabrotica virgifera virgifera*). *Sci. Rep.* **6**, 27860; doi: 10.1038/srep27860 (2016).

## Supplementary Material

Supplementary Information

## Figures and Tables

**Table 1:** Proportion of third instar larvae recovered from field populations and control populations on each of eight maize hybrids.

Hybrid	Control populations	Field populations
Non-Bt near isoline^1^	0.41 ± 0.10 (5)	0.44 ± 0.05 (6)
Cry3Bb1 maize^1^	0.50 ± 0.50 (2) ns	0.23 ± 0.05 (6)*
Non-Bt near isoline^2^	0.52 ± 0.12 (5)	0.33 ± 0.04 (6)
Cry34/35Ab1 maize^2^	0.00 ± 0.00 (2)*^a^	0.00 ± 0.00 (2)**^a^
Non-Bt near isoline^3^	0.47 ± 0.05 (5)	0.34 ± 0.10 (6)
mCry3A maize^3^	0.10 ± 0.10 (5)*	0.42 ± 0.06 (6) ns
eCry3.1Ab maize^3^	------------- (0)^b^	0.31 ± 0.07 (6) ns
mCry3A + eCry3.1Ab maize^3^	0.00 ± 0.00 (2)**^a^	0.46 ± 0.11 (6) ns

Hybrid families are indicated by superscripted numbers following each hybrid category. In total, three hybrid families are tested: ^1^Cry3Bb1 maize and its non-Bt near isoline; ^2^Cry34/35Ab1 maize and its non-Bt near isoline; ^3^mCry3A maize, eCry3.1Ab maize, mCry3A + eCry3.1Ab maize, and a non-Bt near isoline. Data are presented as mean values ± standard error. Numbers in parentheses indicate the number of populations (i.e., sample sizes) that were used to calculate these values. Within a population type (e.g., control populations), the proportion of third instar larvae from a Bt maize hybrid (e.g., Cry3Bb1 maize) was compared to the non-Bt near isoline using a t-test, with *P < 0.05, **P < 0.001, NS = no significant difference from non-Bt near isoline. ^a^Because of unequal variance, a Satterthwaite correction was applied to the t-test. ^b^In one case, a t-test was not conducted, and this occurred because a proportion third instar larvae could not be calculated due to a lack of survival on eCry3.1Ab maize by the control populations, as indicated by a dashed line (--------).
