# Supplementary material for: Broad-spectrum resistance to Bacillus thuringiensis toxins by western corn rootworm (Diabrotica virgifera virgifera)
Source: Sci Rep. 2016 Jun 14;6:27860. doi: 10.1038/srep27860 (PMC4906537; doi:10.1038/srep27860)
Supplement: Supplementary Information [file srep27860-s1.pdf]

Broad-Spectrum Resistance to *Bacillus thuringiensis* Toxins by Western Corn Rootworm (*Diabrotica virgifera virgifera*)

Siva R. K. Jakka, Ram B. Shrestha and Aaron J. Gassmann

	Proportion Survival by Type of Maize[a,b]							
Population[c]	1	2	3	4	5	6	7	8
P1	25±03<br>(8)	21±04<br>(8)	21±04<br>(8)	00±00<br>(8)	30±08<br>(8)	13±03<br>(8)	10±04<br>(8)	04±02<br>(8)
P2	30±06<br>(9)	17±05<br>(9)	20±05<br>(9)	00±00<br>(9)	18±06<br>(9)	15±04<br>(9)	09±03<br>(9)	13±02<br>(9)
P3	20±07<br>(9)	31±05<br>(9)	32±05<br>(9)	00±00<br>(9)	27±07<br>(9)	35±05<br>(9)	19±05<br>(9)	19±04<br>(9)
P4	19±05<br>(9)	27±06<br>(9)	15±04<br>(9)	05±02<br>(9)	30±06<br>(9)	26±05<br>(9)	06±02<br>(9)	08±03<br>(9)
P5	38±05<br>(4)	15±04<br>(4)	28±04<br>(8)	02±02<br>(8)	30±07<br>(8)	18±04<br>(8)	13±05<br>(8)	14±04<br>(8)
P6	24±04<br>(9)	19±04<br>(9)	24±03<br>(9)	00±00<br>(9)	33±07<br>(9)	32±05<br>(9)	19±04<br>(9)	20±03<br>(9)
C1	19±03<br>(8)	00±00<br>(8)	17±03<br>(4)	00±00<br>(4)	21±08<br>(4)	08±03<br>(4)	00±00<br>(4)	00±00<br>(4)
C2	20±04<br>(8)	01±01<br>(8)	23±03<br>(8)	00±00<br>(8)	32±04<br>(8)	02±01<br>(8)	00±00<br>(8)	01±01<br>(8)
C3	29±03<br>(8)	00±00<br>(8)	23±05<br>(8)	00±00<br>(8)	29±05<br>(8)	07±02<br>(8)	00±00<br>(8)	00±00<br>(8)
C4	27±07<br>(9)	00±00<br>(7)	30±09<br>(9)	01±01<br>(9)	49±08<br>(9)	04±02<br>(9)	00±00<br>(9)	00±00<br>(9)
C5	29±06<br>(8)	02±01<br>(8)	26±05<br>(8)	01±01<br>(8)	31±07<br>(8)	02±01<br>(8)	00±00<br>(8)	03±03<br>(8)

Supplementary Table S1. Summary statistics for each combination of population by hybrid tested in bioassays. [a]Numeric values indicate the type of maize: 1 = non-Bt near isoline to Cry3Bb1 maize, 2 = Cry3Bb1 maize, 3 = non-Bt near isoline to Cry34/35Ab1 maize, 4 = Cry34/35Ab1 maize, 5 = non-Bt near isoline to maize with either mCry3A or eCry3.1Ab, 6 = mCry3A maize, 7 = eCry3.1Ab maize, 8 = maize pyramided with mCry3A and eCry3.1Ab. [b]Data are presented an mean proportion survival ± standard error of the mean, with sample size (i.e., number of bioassays conducted) given in parentheses. [c]Populations denoted by a P (i.e., field populations) correspond to Fig. 1. and populations denoted by a C (i.e., control populations) correspond to the following sites: C1 = Phelps Co., NE; C2 = Potter Co., SD; C3 = York Co., NE; C4 = Centre Co., PA; C5 = Finney Co., KS (see Methods for more details).
